# Green Tea Extract Rich in Epigallocatechin-3-Gallate Prevents Fatty Liver by AMPK Activation via LKB1 in Mice Fed a High-Fat Diet

**DOI:** 10.1371/journal.pone.0141227

**Published:** 2015-11-04

**Authors:** Aline B. Santamarina, Juliana L. Oliveira, Fernanda P. Silva, June Carnier, Laís V. Mennitti, Aline A. Santana, Gabriel H. I. de Souza, Eliane B. Ribeiro, Cláudia M. Oller do Nascimento, Fábio S. Lira, Lila M. Oyama

**Affiliations:** 1 Departamento de Fisiologia—Universidade Federal de São Paulo, São Paulo, Brazil; 2 Programa de Pós-Graduação Interdisciplinar em Ciências da Saúde—Universidade Federal de São Paulo, Santos, Brazil; 3 Laboratório de Movimento Humano—Universidade São Judas Tadeu, São Paulo, Brazil; 4 Exercise and Immunometabolism Research Group, Department of Physical Education, Universidade Estadual Paulista, UNESP, Presidente Prudente, SP, Brazil; University of Basque Country, SPAIN

## Abstract

Supplementation with epigallocatechin-3-gallate has been determined to aid in the prevention of obesity. Decaffeinated green tea extract appears to restore a normal hepatic metabolic profile and attenuate high-fat diet (HFD)-induced effects, thereby preventing non-alcoholic fatty liver disease in mice. Mice were maintained on either a control diet (CD) or HFD for 16 weeks and supplemented with either water or green tea extract (50 mg/kg/day). The body mass increase, serum adiponectin level, and lipid profile were measured over the course of the treatment. Furthermore, the AMPK pathway protein expression in the liver was measured. From the fourth week, the weight gain in the CD + green tea extract (CE) group was lower than that in the CD + water (CW) group. From the eighth week, the weight gain in the HFD + water (HFW) group was found to be higher than that in the CW group. Moreover, the weight gain in the HFD + green tea extract (HFE) group was found to be lower than that in the HFW group. Carcass lipid content was found to be higher in the HFW group than that in the CW and HFE groups. Serum analysis showed reduced non-esterified fatty acid level in the CE and HFE groups as compared with their corresponding placebo groups. Increased adiponectin level was observed in the same groups. Increased VLDL-TG secretion was observed in the HFW group as compared with the CW and HFE groups. Increased protein expression of AdipoR2, SIRT1, pLKB1, and pAMPK was observed in the HFE group, which explained the reduced expression of ACC, FAS, SREBP-1, and ChREBP in this group. These results indicate that the effects of decaffeinated green tea extract may be related to the activation of AMPK via LKB1 in the liver of HFD-fed mice.

## Introduction

It is well known that a high-fat diet (HFD) rich in saturated fat and low in dietary fiber can lead to obesity. Obesity, as a systemic and multifactorial disease, causes more damage than just adipocyte hypertrophy [1,2]. Charlton et al. [3] considered that non-alcoholic fatty liver disease (NAFLD) is the hepatic manifestation of obesity and predicted that within 20 years, non-alcoholic steatohepatitis (NASH) will be the leading cause of liver cirrhosis requiring a transplant.

Insulin resistance in visceral adipose tissues in obesity has been shown to lead to an increased activation of the lipolytic signaling pathway [4,5], which further enhances non-esterified fatty acid (NEFA) uptake into the liver. The high hepatic influx of NEFA increases the secretion of very low density lipoproteins (VLDLs) and apolipoprotein B in the circulation, contributing to an increased hepatic glucose production by gluconeogenesis [6] and the activation of the *de novo* lipogenesis pathway [7]. NEFA overload induces an increase in triacylglycerol (TAG) level, exceeding the capacity of VLDL-TG synthesis, thereby promoting TAG accumulation in hepatocytes and contributing to the initiation of NAFLD [8,9].

Research on HFD animal models have shown that AMP-activated protein kinase (AMPK) phosphorylation via liver kinase B1 (LKB1) may be regulated by dietary patterns [10,11]. Furthermore, HFD *per se* may reduce adiponectin level, resulting in the reduction of the phosphorylation of AMPK, which can be activated by this adipokine [12,13]. LKB1 phosphorylation appears to be required for AMPKα activation. The role of adiponectin in LKB1 activation is controversial because a study [14] demonstrated its stimulation, whereas another study [10] did not. Further studies are needed to understand these mechanisms.

The complex formed by LKB1 and AMPK plays a key role in the regulation of hepatic fatty acid metabolism [15]. This complex is activated via phosphorylation. Several molecules activate LKB1 in the liver; one of them is SIRT1 [16]. Studies have shown that when activated by phosphorylation, this process regulates pLKB1 upstream phosphorylation of AMPK [17,18]. Activated pAMPK has the ability to modulate lipogenesis. The phosphorylation of AMPK leads to the phosphorylation and inactivation of acetyl-CoA carboxylase (ACC), which is an important regulatory enzyme in the synthesis of fatty acids by *de novo* lipogenesis [19,20]. ACC catalyzes the conversion of acetyl-CoA to malonyl-CoA via fatty acid synthase (FAS), an enzyme used in the synthesis of fatty acids. The inhibition of ACC by pAMPK reduces substrate flow for FAS, leading to a decrease in the activity of FAS [21].

Moreover, the NAFLD model demonstrated that AMPKα is a negative regulator of sterol element-binding protein 1-c (SREBP 1-c) and carbohydrate response element-binding protein (ChREBP). The increased phosphorylation of AMPKα appears to lead to a decrease in nuclear SREBP 1-c and ChREBP levels. This suggests the existence of a counter regulatory relationship between AMPKα/SREBP 1-c and ChREBP [13,22,23].

The effects of green tea (*Camellia sinensis*) in the prevention and treatment of obesity have been examined [24,25]. Epigallocatechin-3-gallate (EGCG) is the most abundant and biologically active catechin in green tea [26]. Studies have demonstrated that EGCG can reduce the risk of obesity and has possible protective effects against liver damage [2,27–29].

Banerjee et al. [18] report that a single dose of green tea extract was capable of increasing hepatic AMPK level and phosphorylation of LKB1, demonstrating the potent effects of green tea on these molecules. Studies with similar bioactive compounds have suggested that EGCG could prevent peripheral insulin resistance induced by the increase in NEFA level and that this protective effect may be associated with the inhibition of oxidative stress and AMPK signaling activation [30–36]. Stimulation of AMPK phosphorylation, which in turn inhibits ACC and FAS activity, could reduce the accumulation of hepatic TAGs.

Considering the lack of studies regarding the effect of decaffeinated green tea extract on the activation of the AMPK via LKB1, the purpose of this study was to investigate the effects of a green tea extract supplement on the activation of enzymes and factors related to hepatic *de novo* lipogenesis, concurrent with VLDL-TG secretion in HFD-fed mice.

## Materials and Methods

### Animal experiments

All animals experiments were performed according to protocols approved by the Experimental Research Committee of Universidade Federal de São Paulo (CEUA n° 975418) respecting the standards established by the Brazilian Guideline for Care and Use of Animals for Scientific Purposes and Teaching imposed by the National Council of Animal Experimentation—CONCEA in 2013.[37]. A total of 54 male Swiss mice at 30 days old were used. To total number of samples, the experimental protocol was performed twice, to prove the replicability of our model.

The mice were maintained in collective polypropylene cages in isolated room with controlled temperature (25± 2°C), humidity (60 ± 5%) and lighting (12-h light/dark cycle) and received water and diet *ad libitum* during all experimental period. After one week of acclimatization, the mice were divided evenly into four groups: Control diet + water (CW) (n = 14); Control diet + green tea extract rich in EGCG (CE) (n = 14); High-fat diet + water (HFW) (n = 13) and High-fat diet + green tea extract rich in EGCG (HFE) (n = 13). The mice were fed control diet (AIN-93) [38] or high-fat diet (AIN-93 adapted) and water, both *ad libitum* during the next 16 weeks (Table 1).

**Table 1:** Composition of experimental diet CD and HFD (AIN-93 modified) [38], growth (G) and maintenance (M).

Ingredients	CD (G/M)	HFD (G/M)
**Cornstarch (%)**	62. 95 / 72.07	40. 92 / 40.87
**Casein (%)**	20. 0 / 14.0	13. 95 / 14.0
**Soybean Oil (%)**	7 0.0 / 4.0	7.0 / 4.0
**Lard (%)**	-	28. 08 / 31.2
**Cellulose (%)**	5	5
**Mixture of Vitamins (%)**	1.0	1.0
**Mixture of Mineral (%)**	3.5	3.5
**L-cystine (%)**	0. 3 / 0.18	0.18 / 0.18
**Cholinebitartrate (%)**	0.25	0.25
**Hydroquinone (g/kg)**	0.014 / 0.008	0.014 / 0.008
**Energy (Kcal/kg)**	3948 / 3.802	5.352 / 5.362

To ensure accurate use of the established dose of green tea extract, administration of green tea was performed by gavage. Thereby the HFE and CE groups received treatment green tea extract (0.1 mL green tea extract 98% EGCG - 50mg/kg/day). To guarantee that all animals pass by the same conditions of stress, manipulation and physical injury, the animals belonging to the placebo group (HFW and CW) were also submitted to gavage received filtered water in equal volume to that given for the groups treated with green tea extract (0.1 mL of water by gavage / day). The animals of placebo groups were weighed once a week, the weight of the green tea extract treatment groups were measured every day during the experimental period to adjust the daily dose to weight.

At the end of the experimental period, the mice were euthanized by beheading in the morning period between eight and ten o'clock, after 12 hours fasting. Blood and liver were collected for analyzes, the hepatic tissues were weighed and all samples were stored at −80°C. All experimental procedures described below were performed using single samples, sample pooling has not been necessary.

### Experimental Procedures

#### Carcass lipid and protein content

The carcass lipid and protein content was determined in all experimental groups. The carcasses were eviscerated, and the retroperitoneal, epididymal and mesenteric white adipose tissue, gastrocnemius muscle and liver were removed. The remaining carcasses were weighed and stored at −20°C. The lipid content was measured as described by Stansbie et al.[39] and standardized using the method described by Oller do Nascimento and Williamson [40]. Briefly, the eviscerated carcass was autoclaved at 120°C for 90 min and homogenized with water at a volume twice the carcass mass. Triplicate aliquots of this homogenate were weighed and digested in 3 mL of 30% KOH and 3 mL of ethanol for ≥2 h at 70°C in capped tubes. After cooling, 2 mL of 12 N H_2_SO_4_was added, and the samples were washed three times with petroleum ether to extract the lipids. The results are expressed as grams of lipid per 100 g of carcass. To measure the protein content, aliquots of the same homogenate (approximately 1 g) were heated to 37°C for 1 h in 0.6 N KOH with constant shaking. After clarification by centrifugation, the protein content was measured using the Bradford assay (Bio-Rad, Hercules, California) with bovine serum albumin as a reference.

#### Serum parameters

The serum glucose, total cholesterol (TC), HDL-cholesterol and triacylglycerol (TAG) were analyzed by colorimetric method using commercial kits (Labtest ®). The serum concentration of adiponectin (R&D Systems) and Non-esterified fatty acids (Zen-bio, Inc.) were also evaluated. LDL-cholesterol level was estimated indirectly by Friedewald equation [LDL-c = total cholesterol-(HDL-c)-(TG/5)][41].

#### *In vivo* VLDL-triacylglycerol secretion

Considering the interfering effects that anesthesia can exert on other parameters that would be evaluated, an exclusive group of mice were treated for this trial to determine the *in vivo* VLDL-TAG secretion from the liver, and these mice were excluded from other analyses. The required sample size was five mice per experimental group (n = 5). After the 16-week intervention period, mice were fasted for 4 h to minimize the contribution by intestinal absorption of serum lipoproteins. They were anesthetized using ketamine (80 mg/kg) and xylazine (10 mg/kg) and infused through the jugular vein a Tyloxapol solution (400 mg/kg, Triton WR 1339, Sigma) dissolved in saline solution (300 mg/mL) and heated at 37°C. Blood samples were collected prior to injection and at again at 30, 60, 90, and 120 min after injection for the determination of TAG level. Hepatic VLDL-TAG secretion was then estimated by calculating the area under the curve (AUC). After the experimental procedure, the animals were euthanized by exsanguination trough the jugular vein access used for collecting blood samples during the experiment.

#### Western blot analysis

Hepatic tissue were homogenized in lyses buffer containing 100mM Tris–HCl (pH 7.5), 1% Triton X-100, 10% sodium dodecyl sulfate (SDS), 10mM EDTA, 100mM sodium fluoride, 10mM sodium pyrophosphate, 10mM sodium orthovanadate, 2.0mM phenylmethylsulphonyl fluoride (PMSF), and 0.1mg aprotinin/mL. The homogenate was centrifuged at 20,800 x *g* for 45 min at 4°C, and the supernatant was collected. The total protein concentration was measured with Bradford Reagent (Bio-Rad Laboratories, Inc.). Proteins in the lysates were electrophoretically separated in 10% SDS polyacrylamide gel and transferred to nitrocellulose membrane. Were used precast gels in 4–15% (Mini-PROTEAN^®^TGX ^TM^ Precast Gels) of Bio-Rad Laboratories, Inc. The membranes were blocked in 1% bovine serum albumin overnight at room temperature, and then incubated overnight with the following primary antibodies: pAMPK α1/2 (sc-33524), AMPK α1/2 (sc-25792), ACCα (sc-30212), FAS (sc-20140), SREBP-1 (sc-8984), ChREBP (sc-21189), SIRT-1 (sc-15404) purchased from Santa Cruz Biotechnology, Inc.(Santa Cruz, CA, USA). The β-tubulin, pLKB1 (S428), pACC (S79), pSREBP-1c (Ser372) antibodies were obtained from Cell Signaling Technology (Beverly, MA, USA). Adiponectin Receptor-2 (AdipoR2) was purchased from ABCAM (Cambridge, UK) and LamininR-67KDa (NBP1-33002) was from Novus Biologicals (Littleton, CO, USA).The membranes were next incubated with horseradish-peroxidase-conjugated secondary antibodies during 1 hour at room temperature. The bands were visualized with enhanced chemiluminescence scanned at UVITec (Cambridge) after adding the ECL reagent (GE Healthcare Bio-Sciences AB, UK), and the intensities of the bands were quantified in ImageJ software (ImageJ, National Institute of Health, Maryland, USA). Performing calculations of each band obtained for analysis in proteins of interest for this study were normalized using β-tubulin levels of the respective membrane individually.

### Statistical Analyses

Data were subjected to quality tests such as Shapiro–Wilk (normality), Levenne (homogeneity), and/or Mauchly (sphericity). In cases of non-sphericity, corrected values F (Greenhouse–Geisser) were used. If necessary, data were standardized according to the Z score. The descriptive analysis was performed using the mean ± SEM. To verify the interactions between groups, we used two-way ANOVA followed by Tukey’s post hoc test. The level of significance was p ≤ 0.05. For statistical analyses, the software SPSS Version 17.0 was used.

## Results

### Effects of green tea extract on the mass gain and body composition

**Fig 1:**
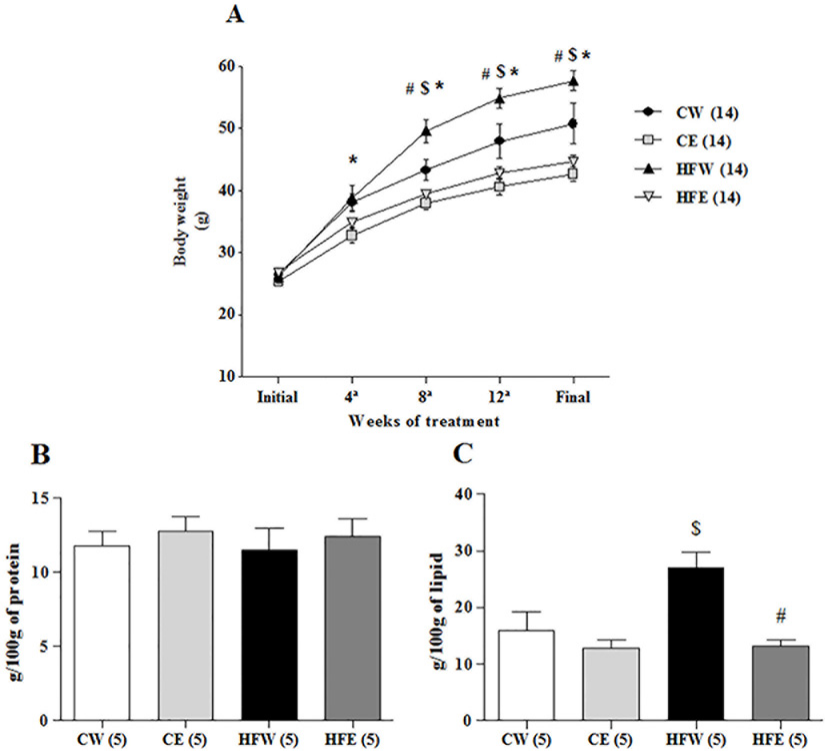
Body composition: body mass gain; lean body mass; and subcutaneous fat accumulation. (A) body mass gain over the 16 weeks of treatment with HFD; (B) lean body mass; (C) subcutaneous fat accumulation obtained from the carcass of different experimental groups. Data are expressed in mean ± s.e.m. *p<0.05 Control diet and EGCG (CE) group versus Control diet and Water (CW) group. $p<0.05 High-fat and Water (HFW) group versus CW group. #p<0.05 High-fat diet and EGCG (HFE) group versus HFW group.

There were no statistically significant differences among the experimental groups when they were initially weighed. Nevertheless, the body weight of mice belonging to the CE group was significantly lower than those belonging to the CW group, starting at the fourth week until the end of experimental treatment (p < 0.001). The effects of HFD on weight gain were observed from the eighth week of experimental treatment, with an increase in mass in the HFW group as compared with the CW group (p < 0.001). Starting at the eighth week, the HFE group also had statistically significant lower weight gain as compared with the HFW and placebo groups (p < 0.001; Fig 1A).

On determining the body composition using the carcass analysis, differences in protein content among the groups were not observed. We observed an increase in the lipid content of carcasses in the HFW group as compared with the CW group (p = 0.005). On the other hand, the green tea extract supplement was able to reduce the lipid content in the HFE group as compared with the HFW group (p < 0.001; Fig 1B and 1C).

### Effects of green tea extract on serum parameters

The serum lipid profiles, NEFA and adiponectin levels are shown in Table 2. Serum TAG (p = 0.049), TC (p = 0.001), and LDL-c (p = 0.010) levels were increased in the HFW group as compared with the CW group. LDL-c level was also reduced in the HFE group as compared with the HFW group (p = 0.011). NEFA level was reduced in the HFE group as compared with the HFW group (p < 0.001) as well as in the CE group as compared with the CW group (p < 0.001). Adiponectin presented a significantly higher level in the HFE group as compared with the HFW group (p = 0.031) and in the CE group as compared with the CW group (p = 0.046). The HDL-c assessment did not show differences among the experimental groups.

**Table 2:** Serum parameter assessed in different groups.

Parameters	CW	CE	HFW	HFE
**TC (mg/dL)**	136.9 ± 2.9	129.7 ± 2.7	163.5 ± 6.2^$^	155.7 ± 4.17
**TAG (mg/dL)**	131.96 ± 3.3	129.89 ± 4.4	163.55 ± 8.5^$^	147.87 ± 7.1
**HDL-c(mg/dL)**	54.28 ± 2.76	52.16 ± 4.04	61.61 ± 3.75	58.77 ± 2.44
**LDL-c (mg/dL)**	63.07 ± 4.18	51.28 ± 9.20	91.39 ± 8.23^$^	78.97 ± 4.48
**NEFA (mM)**	4.67 ±0.27	2.97 ±0.14^*^	4.89 ±0.38	3.19 ±0.15^#^
**Adiponectin(μg/mL)**	8.38 ± 0.47	10.38 ± 0.66^*^	6.90 ± 1.01	8.73 ± 0.73^#^

Data are expressed in mean±s.e.m.

^*^p<0.05 Control diet and EGCG (CE) group versus Control diet and Water (CW) group (n = 14).

^$^p<0.05 High-fat and Water (HFW) group versus CW group (n = 13–14).

^#^p<0.05 High-fat diet and EGCG (HFE) group versus HFW group (n = 13).

### Effect of green tea extract on VLDL-TG secretion

**Fig 2:**
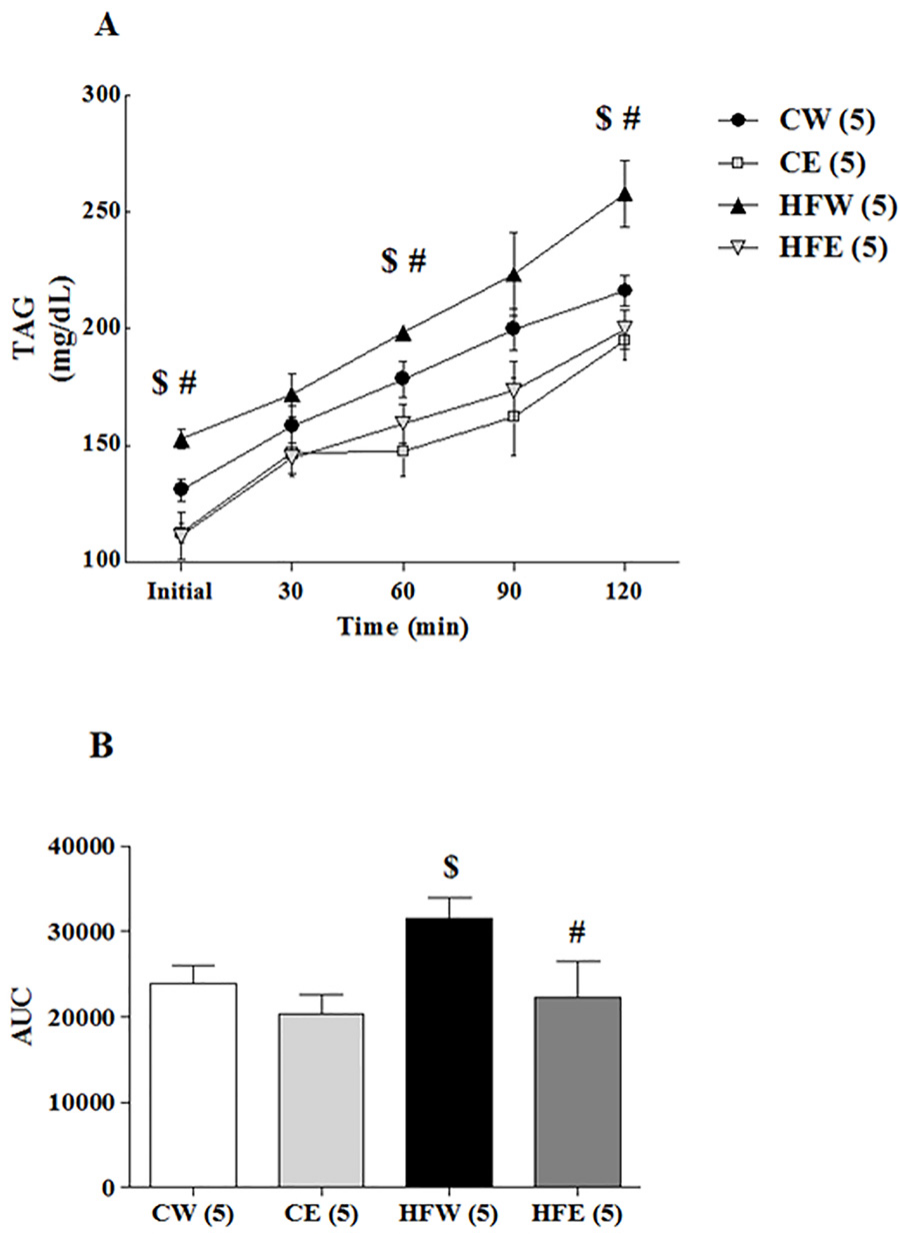
*In vivo* triacylglycerol production rate. After 16 week of treatment. After 16 week of treatment, Triton WR1339 (400 mg/kg BW) was administered intravenously to 4 hours fasted mice. Samples collected at time 0, 30, 60, 90 and 120 min after injection. Plasma samples from each time point were used to determine plasma triacylglycerol levels over time (A). Triacylglycerol production (B) was estimated by calculated the AUC. Data are expressed in mean ± s.e.m.^*^p<0.05 Control diet and EGCG (CE) group versus Control diet and Water (CW) group. ^$^p<0.05 High-fat and Water (HFW) group versus CW group. ^#^p<0.05 High-fat diet and EGCG (HFE) group versus HFW group.

High-fat feeding in mice increases hepatic VLDL-TG secretion, and in the study we tested whether green tea extract can lower the secretion of VLDL-TG. The TAG concentration over time increased in the HFW group as compared with the CW and HFE groups at baseline (p = 0.037 and 0.019, respectively), 60 min (p = 0.028 and p = 0.033, respectively), and 120 min (p = 0.042 and p < 0.001, respectively) after tyloxapol injection. The AUC analysis affirmed the differences in the HFW group compared with the CW group (p = 0.012) and HFE group (p = 0.003; Fig 2A and 2B).

### Effect of green tea extract on liver protein expression

**Fig 3:**
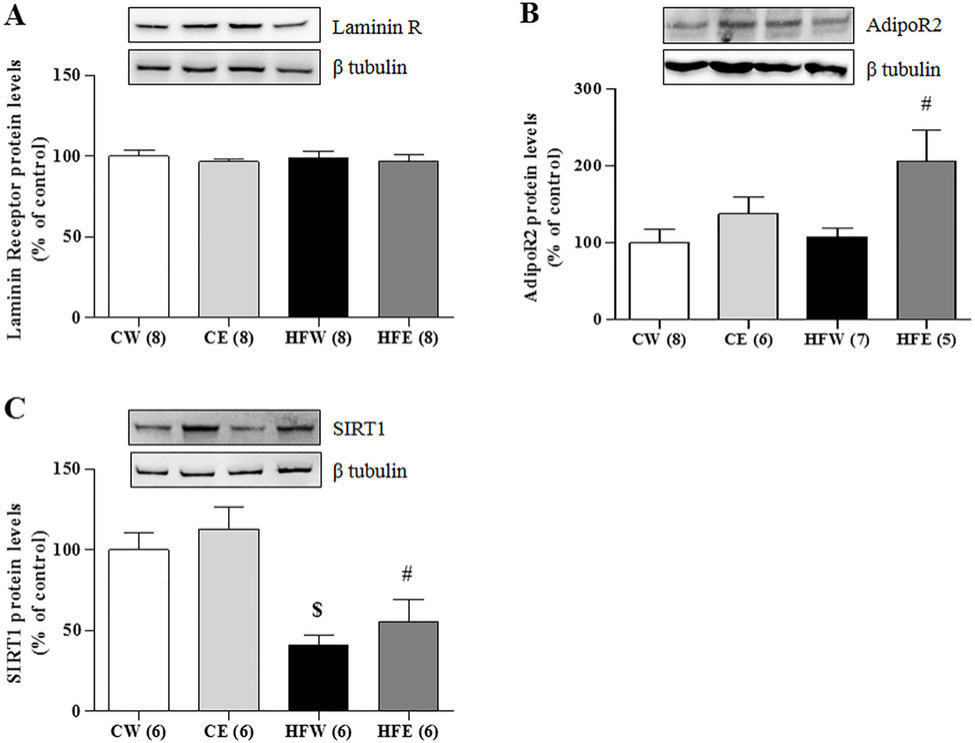
Liver protein expression in different experimental groups of Laminin R, AdipoR2 and SIRT1. Western Blotting analysis of protein expression in the liver on different experimental groups of membrane receptors (A) Laminin Receptor, (B) AdipoR2, and (C) SIRT1. Image shows demonstrative bands of the analyzed proteins and respective housekeeping protein (β-tubulin) in liver. Data are expressed in mean ± s.e.m. ^*^p<0.05 Control diet and EGCG (CE) group versus Control diet and Water (CW) group. ^#^p<0.05 High-fat diet and EGCG (HFE) group versus HFW group. ^$^p<0.05 HFW versus CW.

**Fig 4:**
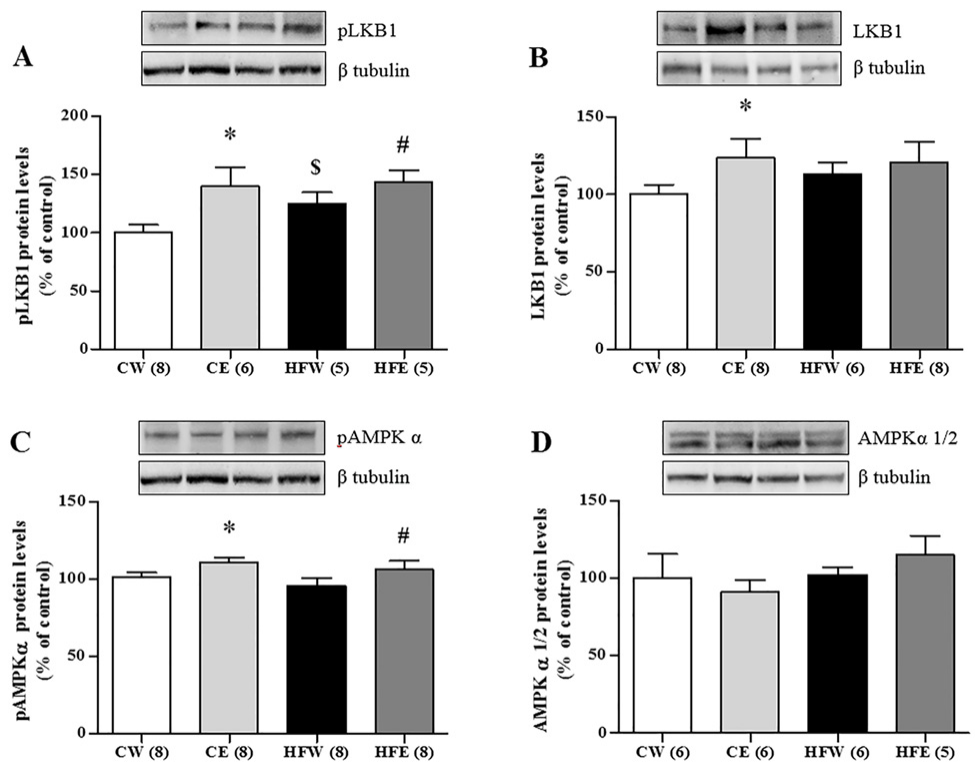
Liver protein expression in different experimental groups of LKB1 / AMPK pathway. Western blotting analysis of protein expression in the liver on different experimental groups of AMPK—LKB1 pathway (A) pLKB1, (B) LKB1, (C) pAMPKα, (D) AMPKα 1/2. Image shows demonstrative bands of the analyzed proteins and respective housekeeping protein (β-tubulin) in liver. Data are expressed in mean ± s.e.m. ^*^p<0.05 Control diet and EGCG (CE) group versus Control diet and Water (CW) group. ^#^p<0.05 High-fat diet and EGCG (HFE) group versus HFW group. ^$^p<0.05 HFW versus CW.

**Fig 5:**
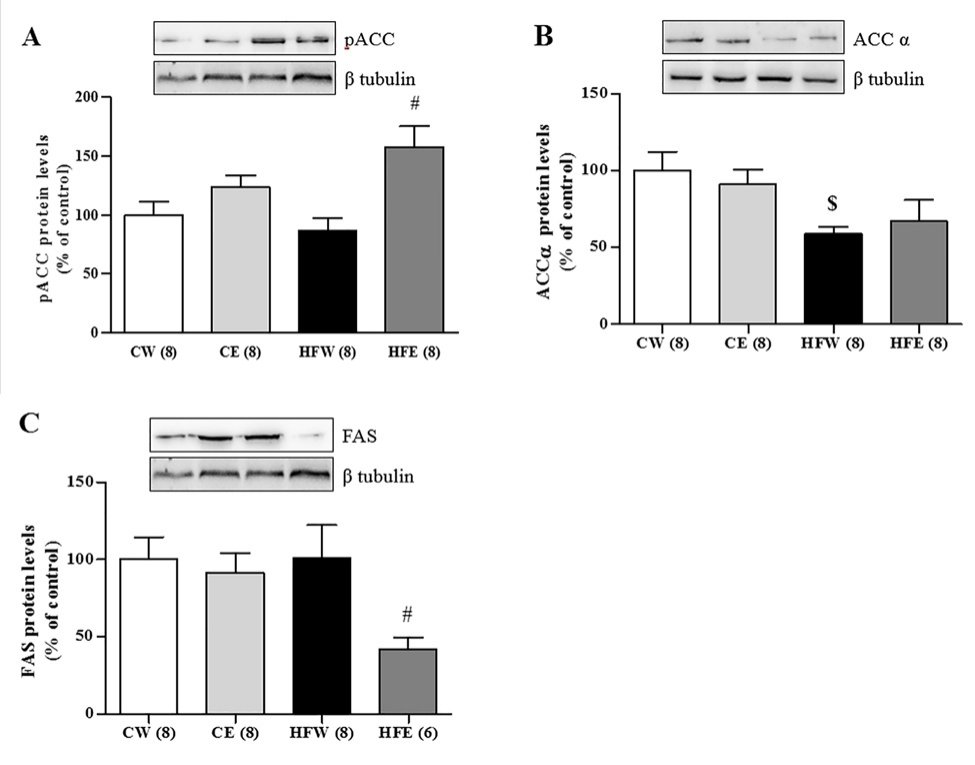
Liver protein expression in different experimental groups of lipogenic enzymes. Western Blotting analysis of protein expression in the liver on different experimental groups of enzymes responsible for synthesis of fatty acids and *de novo* lipogenesis. (A) pACC, (B) ACCα and (C) FAS. Image shows demonstrative bands of the analyzed proteins and respective housekeeping protein (β-tubulin) in liver. Data are expressed in mean ± s.e.m. ^*^p<0.05 Control diet and EGCG (CE) group versus Control diet and Water (CW) group. ^#^p<0.05 High-fat diet and EGCG (HFE) group versus HFW group. ^$^p<0.05 HFW versus CW.

**Fig 6:**
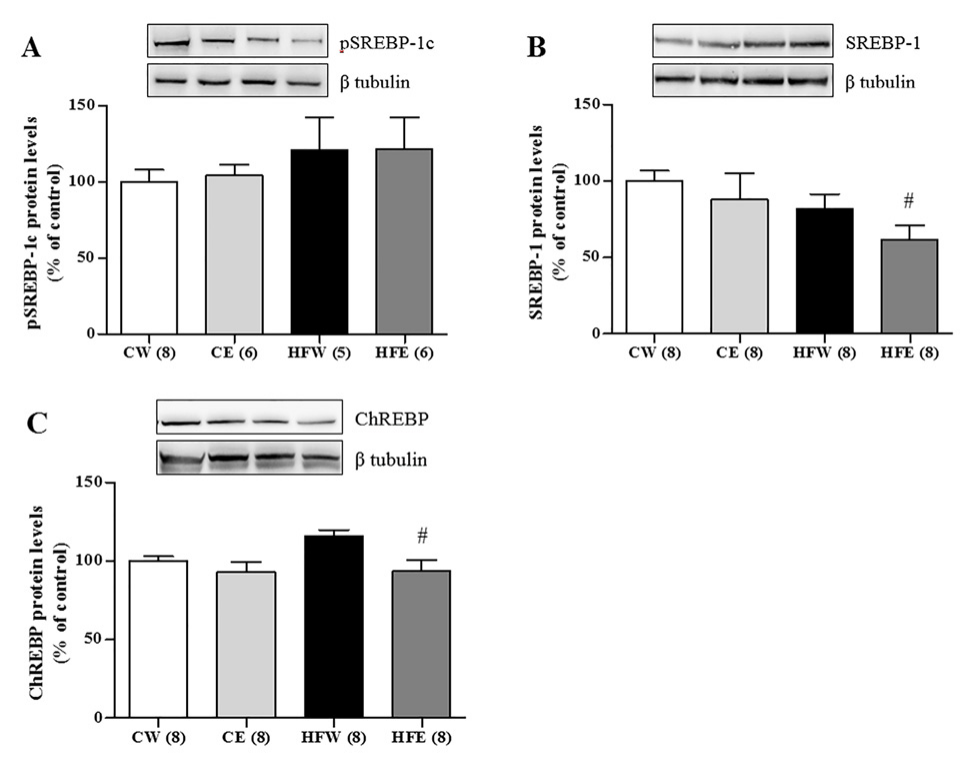
Liver protein expression of nuclear transcription factors of lipids metabolism in different experimental groups. Western Blotting analysis of protein expression in the liver on different experimental groups of nuclear transcription factors involved in promotion of enzymes responsible for synthesis of fatty acids and *de novo* lipogenesis. (A) pSREBP-1c, (B) SREBP-1, (C) ChREBP. Image shows demonstrative bands of the analyzed proteins and respective housekeeping protein (β-tubulin) in liver. Data are expressed in mean ± s.e.m. ^*^p<0.05 Control diet and EGCG (CE) group versus Control diet and Water (CW) group. ^#^p<0.05 High-fat diet and EGCG (HFE) group versus HFW group. ^$^p<0.05 HFW versus CW.

The protein expression of the laminin membrane receptors did not differ among the groups, but AdipoR2 expression in the liver was increased in the HFE group as compared with the HFW group (p = 0.006). SIRT1 expression was reduced in the HFW group compared as with the CW group (p = 0.004). Green tea extract improved SIRT1 expression in the HFE group as compared with the HFW group (p = 0.004; Fig 3A–3C). Green tea extract treatment also caused a specific increase in the phosphorylation of LKB1 in the HFE group (p = 0.047) as compared with the HFW group as well as in the CE (p = 0.034) and HFW (p = 0.027) groups in relation to the CW group. LKB1 was also elevated individually in the CE group compared with the CW group (p = 0.046). The activation through pAMPKα 1/2 increased in the CE group as compared with the CW group (p = 0.037) and in the HFE group (p = 0.034) as compared with the HFW group. The total fraction of AMPKα 1/2 did not differ among the groups (Fig 4A–4D). The lipogenic enzyme ACCα in its active form (not phosphorylated) was activated and higher in the HFW group as compared with the HFE group (p = 0.001) and the enzyme pACC in its inactive form (phosphorylated) was higher in the HFE group as compared with the HFW group (p = 0.017; Fig 5A and 5B). FAS and ChREBP were reduced in the HFE group as compared with the HFW group (p = 0.012 and p = 0.008, respectively). The total protein expression SREBP-1c that represents the active isoform was lower in the HFE group as compared with the HFW group (p = 0.007); on the other hand, the pSREPB-1c that represents the inactive isoform (phosphorylated) did not change among the groups (Figs 5C and 6A–6D).

## Discussion

The main contribution of this study was new, additional information suggesting that decaffeinated green tea extract rich in EGCG may exert reduction on the *de novo* lipogenesis and secretion of VLDL. Our data suggest that green tea extract seems to have an influence on the increase of adiponectin production, which in turn could represent one mechanism of AMPK activation by LKB1. This may reduce the level of *de novo* lipogenesis and secretion of VLDL, suggesting a potential therapy for the prevention of liver damage in HFD-associated obesity.

After analyzing the data regarding body composition in this study, we can see the effectiveness of our obesity model. The *ad libitum* supply of HFD led to an increase in weight gain as well as an increase in the carcass fat content, and this was maintained throughout the treatment until the end of the experimental period (Fig 1C). Based on these parameters, it was also possible to observe the activity of green tea extract in reducing the effects of obesity (Fig 1A). Our results are widely supported by previous studies in which the same effects were observed following the administration of HFDs [5,42–46]. Similar effects of EGCG supplementation in preventing the deleterious effects of HFD have also been reported [24,25,47–52].

Some authors have described anti-nutritional effects of the components of green tea, including EGCG, in inhibiting the absorption of nutrients [53–55]. HFD, in some situations, has also been shown to cause sarcopenia [56–59]. The gastrocnemius relative weight (data not shown) and analysis of protein concentrations of carcasses showed that there was no depletion of lean body mass among the groups as compared with the control (Fig 1B). The absence of obesity-related sarcopenia means that our HFD model met the physiological and nutritional requirements for the maintenance of lean body mass. It also shows that the reduction in weight gain observed with green tea extract supplementation is not related to anti-nutritional factors of catechins or lack of nutrient absorption, which can be verified by the normal lean body mass seen in all experimental groups.

Another metabolic characteristic linked to obesity occurs in modifying serum parameters. For the classification of the metabolic syndrome, changes in serum lipid profile are used in addition to weight gain [60]. It is a common initiator of dyslipidemia: an increase in TAG, TC and LDL-c levels and a decrease in HDL-c level. The results based on these parameters show that our experimental model of obesity was effective in inducing obesity-linked metabolic changes, confirmed by a worsening of the TAG, TC, and LDL-c levels; however, there was no change in HDL-c level (Table 2).

Being multifactorial diseases, obesity and the metabolic syndrome have systemic, deleterious effects. Of all the comorbidities attributed to obesity, NAFLD is the only hepatic manifestation of obesity. Because the liver is an organ of vital importance for the maintenance of body homeostasis, it is extremely important to undertake measures to prevent and combat the initiation of NAFLD [2,3].

With increasing visceral adiposity, there is increased portal NEFA delivery to the liver. It has been demonstrated that the fraction of NEFA delivered to the liver from visceral fat is positively correlated to the visceral fat area, and it is approximately 5%–10% in normal-weight subjects and 20%–30% in obese subjects [61]. Hepatic uptake of NEFA is proportional to the rate of delivery. In the liver, NEFA is either oxidized or esterified to form TAGs. The TAG is stored in the cytosol prior to being incorporated into VLDLs and then secreted. When TAG production exceeds NEFA oxidation and VLDL production and secretion, TAGs accumulate in the liver, resulting in the development of NAFLD [62,63]. In our results, NEFA level was increased in the HFW group, and the green tea extract supplementation was able to reduce NEFA level, contributing to the prevention of NAFLD development (Table 2). When *in vivo* VLDL-TG secretion was analyzed (Fig 2), it was clear that green tea extract reduced the secretion rate of VLDL-TG (in agreement with the NEFA data) and decreased the amounts of this lipoprotein in the blood circulation.

After reviewing the literature, we had indications that Laminin Receptor 67 kDa could be increased by EGCG [64–68]. However, our results showed no change in protein expression of this receptor in the liver. This finding suggests that the dose chosen for our experimental treatment was physiologically adequate to exert beneficial effects without stimulating the increase of membrane receptor synthesis because this receptor can be found in large quantities in cancer cells [69].

Over the last few years, several authors have described that green tea polyphenols and isolated EGCG are effective in raising serum adiponectin level and protein expression of its receptors (AdipoR1 and AdipoR2) in humans and HFD-fed mice [25,70–72]. Adiponectin gene expression is mainly regulated by the PPARγ nuclear transcription factor. PPARγ binds directly to the functional element PPAR-responsive element (PPRE) in the adiponectin promoter and increases the transcription of the adiponectin gene [73]. Tian et al. [74] demonstrated that green tea polyphenols can inhibit the activation of ERK1/2, increase the expression of PPARγ, and reduce the phosphorylation of PPARγ. Based on these findings, we suggest that in our study, green tea extract was responsible for the increase in the adiponectin level (Table 2).

Increased adiponectin and AdipoR2 levels have been shown to correlate with the activation of SIRT1 [75–77]. Civitarese et al. [78] demonstrated that adiponectin significantly increased SIRT1 protein expression but had no effect on SIRT1 mRNA, an indicator of post-transcriptional regulation. It was also demonstrated that knockout adiponectin receptors (-R1 and -R2 isoforms) reduced the amount of SIRT1 protein in primary human myotubes, indicating that adiponectin could directly upregulate SIRT1. These findings support our hypothesis that adiponectin, through its most abundant receptor in the liver (AdipoR2), stimulated the activation of SIRT1 in the HFE group (Fig 3B and 3C).

SIRT1 appears to activate AMPKα via a LKB1-dependent pathway in HFD. This has been reported in human hepatocytes [16] and reinforced by our study. Results suggests that AdipoR2 activates SIRT1, which in turn is responsible for the deacetylation of Lys48 residues on nuclear LKB1 [79,80]. When activated LKB1 migrates from the hepatocyte nucleus to the cytoplasm, where it induces phosphorylation of the AMPK α subunit [81]. The AMPK heterotrimeric molecule conformation is composed of three subunits, which exert different biological functions. It presents two regulatory subunits (AMPKβ and γ) and the catalytic subunit AMPKα is mainly responsible for the AMPK activity in rodent liver [82]. LKB1 can specifically phosphorylate the catalytic subunit (AMPKα 1/2), as shown in our results, particularly in the HFD group (Fig 2A–2D). The subunits AMPKα 1a and AMPKα 2 were the most discussed in the literature concerned with the effectiveness of AMPK activation [83,84].

ChREBP and SREBP 1-c are transcription factors that regulate the expression of hepatic enzymes involved in lipogenesis. These factors can bind to the transcription promoter genes of lipogenic proteins such as ACC, FAS, and fatty acid converting enzymes such as stearoyl CoA desaturase (SCD1) and fatty acid elongases such as fatty acid elongase 6 [85,86]. Both ChREBP and SREBP 1-c remain in the cytoplasm attached to an inactive phosphate group. The dephosphorylated (active) form has shown the ability to translocate to the nucleus and stimulate the synthesis of lipogenic enzymes [80,87]. Some studies reported that activated AMPK has a role in maintaining the phosphorylated SREBP1c and CHREBP forms in the cytoplasm and preventing them from reaching the nucleus [13,22,23,32,88,89]. These results agree with ours, wherein there was a reduction in the active forms of ChREBP and SREBP1c in the HFE group as compared with the HFW group. This led to reduced protein expression of lipogenic enzymes in the liver (Fig 6A–6C).

Activated AMPK demonstrated the ability to reduce the activity of the lipogenic enzymes, ACC and FAS [90]. ACC catalyzes the conversion of acetyl-CoA to malonyl-CoA, which is used in the synthesis of fatty acid *de novo* lipogenesis [85]. In our study, when phosphorylated, ACC was in its inactive form and we clearly observed that supplementation with green tea extract increased pACC level as well as reduced the FAS level, indicating that there was a reduction in fatty acid synthesis by the *de novo* lipogenesis pathway (Fig 5A–5C).

## Conclusion

**Fig 7:**
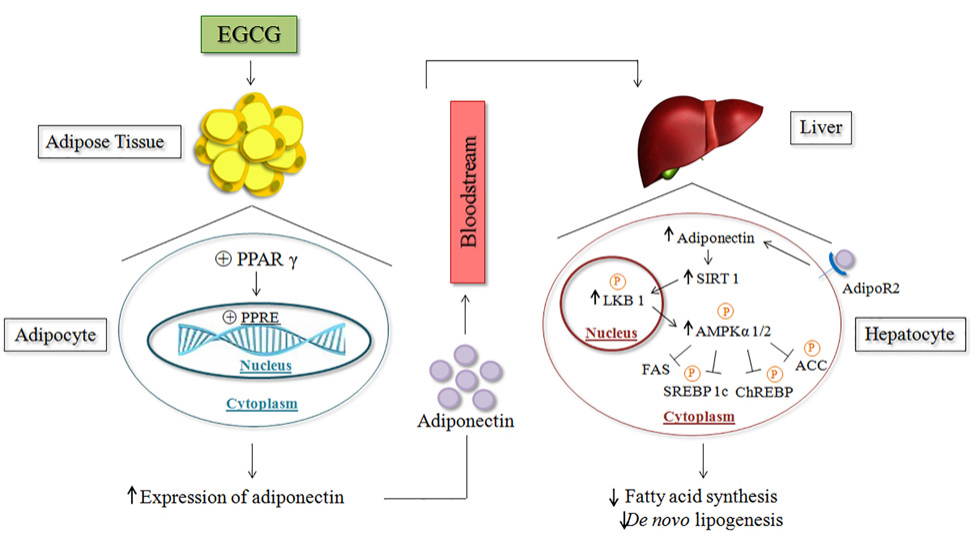
EGCG through adiponectin and LKB1 / AMPK pathway on lipid metabolism—possible mechanism. Statement from mechanism by which EGCG promotes increase in adiponectin and performs activation on the signaling of AMPK via LKB1 inhibiting mediators responsible for the synthesis of fatty acids and *de novo* lipogenesis. (Liver and DNA- from *FreeDigitalPhotos*.*net)*.

Overall, our results indicate that green tea extract supplementation was able to improve hepatic metabolism and reduce NEFA uptake into the liver; this led to a decrease in hepatic VLDL-TG secretion, decreased lipid synthesis, and improvement of adiponectin in HFD-fed mice. Highlighting its beneficial effects, it was noted that green tea extract supplementation has also been shown to stimulate the activation of the AMPK via LKB1 through adiponectin in HFD-fed mice and to regulate essential enzymes involved in the *de novo* lipogenesis pathway in the liver. This beneficial effect may contribute to the prevention and treatment of NAFLD (Fig 7).
